# All-Trans Retinoic Acid Induces Proliferation, Survival, and Migration in A549 Lung Cancer Cells by Activating the ERK Signaling Pathway through a Transcription-Independent Mechanism

**DOI:** 10.1155/2015/404368

**Published:** 2015-10-18

**Authors:** Reyna Sara Quintero Barceinas, Alejandro García-Regalado, Elena Aréchaga-Ocampo, Nicolás Villegas-Sepúlveda, Claudia Haydée González-De la Rosa

**Affiliations:** ^1^Departamento de Ciencias Naturales, Universidad Autónoma Metropolitana, Unidad Cuajimalpa, Avenida Vasco de Quiroga 4871, 05348 Colonia Santa Fe Cuajimalpa, DF, Mexico; ^2^Facultad de Enfermería, Universidad Autónoma de San Luis Potosí, Avenida Niño Artillero 130, 78240 Zona Universitaria, SLP, Mexico; ^3^Departamento de Biomedicina Molecular, Centro de Investigación y de Estudios Avanzados del IPN, Avenida Instituto Politécnico Nacional 2508, 14740 Colonia San Pedro Zacatenco, DF, Mexico

## Abstract

All-trans retinoic acid (ATRA) has been used as an antineoplastic because of its ability to promote proliferation, inhibition, and differentiation, primarily in leukemia; however, in other types of cancer, such as lung cancer, treatment with ATRA is restricted because not all the patients experience the same results. The ERK signaling pathway is dysregulated in cancer cells, including lung cancer, and this dysregulation promotes proliferation and cell invasion. In this study, we demonstrate that treatment with ATRA can activate the ERK signaling pathway by a transcription-independent mechanism through a signaling cascade that involves RAR*α* and PI3K, promoting growth, survival, and migration in lung cancer cells. Until now, this mechanism was unknown in lung cancer cells. The inhibition of the ERK signaling pathway restores the beneficial effects of ATRA, reduces proliferation, increases apoptosis, and blocks the cell migration process in lung cancer cells. In conclusion, our results suggest that the combination of ATRA with ERK inhibitor in clinical trials for lung cancer is warranted.

## 1. Introduction

Lung cancer is the second most common cancer and is the leading cause of cancer mortality worldwide for both men and women [1]. It is estimated that lung cancer accounts for 1.6 million newly registered cases of cancer and for 1.37 million cancer deaths annually [2]. Most patients will present with incurable disease with a poor life expectancy after standard therapy treatments [3]. In recent years, new treatments with novel action mechanisms have been explored for advanced lung cancer, including retinoid administration [4]. All-trans retinoic acid (ATRA) is a retinoid and a promising agent in the treatment of cancer due to its ability to inhibit proliferation and induce apoptosis in many* in vivo* and* in vitro* assays on tumor cells [5]. ATRA is frequently used as differentiation inductor both in leukemia and in solid tumors such as neuroblastoma [6]. The biological effects of ATRA are achieved through binding to RAR nuclear receptors [7]. RAR can form heterodimers with other nuclear receptor types including RXR; this association is needed to enable the protein complexes to bind to retinoic acid responsive elements (RARE) located in the promoters of their target genes and to induce transcription [8]. There is also evidence that ATRA can activate survival pathways, which are mechanisms that enable cancer cells to become resistant to ATRA treatment. ATRA may also directly regulate the activation of some kinase signaling pathways by so-called nongenomic mechanisms, which do not involve a transcriptional response [9], such as retinoylation [10].

We previously reported that activation of Akt blocks the transcriptional effects of ATRA, promotes invasion and cell survival, and confers resistance to retinoic acid treatment in lung cancer cells [11]. We proposed that survival pathway activation in response to retinoid treatment might be a resistance mechanism of lung cancer cells. The short-term activation of other signaling pathways by ATRA has also been reported. In PC12 and bronchial epithelial cells, there have been reports that ATRA activated ERK within the first 30 minutes after treatment by a mechanism independent of RARs function [12]. However, in neuroblastoma cells, ERK activation involves retinoid binding to RAR and activation of PI3K independent of gene transcription [13]. In neurons, ATRA triggers the activation of ERK within 10 minutes and is mediated by an RAR-dependent mechanism [14]. Contrary, ATRA mediated ERK inactivation in human scleral fibroblasts [15]. Therefore, activation or inhibition of ERK by ATRA is dependent on the cellular context and tissue type. In this report, we demonstrated that ATRA activates ERK signaling in the A549 cell line by a mechanism independent of gene transcription. ERK activation promotes cell survival and migration, blocking the anticancer effect of ATRA. Such activation results in the development of retinoids resistance in the lung cancer cells.

## 2. Materials and Methods

### 2.1. Cell Lines and Treatments

A549 cells were routinely grown in DMEM/F12 medium supplemented with 10% fetal bovine serum (FBS), 100 IU/mL penicillin, and 100 *μ*g/mL streptomycin at 37°C in a 5% CO_2_ atmosphere. ATRA, the PI3K kinase inhibitor (wortmannin), and the RAR*α* antagonist (Ro 41-5253) were purchased from Sigma-Aldrich, Inc. (St. Louis, MO, USA). The MEK inhibitor (PD98059) was purchased from Enzo Life Science, Inc. (Farmingdale, NY, USA), and the pan-RAR-antagonist (AGN193109) was purchased from Santa Cruz Biotechnology, Inc. (Santa Cruz, CA, USA). The different compounds were dissolved in dimethyl sulfoxide and added to the culture medium at the indicated concentrations.

### 2.2. Western Blot

Whole-cell extracts were obtained by lysis of the A549 cells in lysis buffer [20 mM Tris–HCl (pH 7.5), 1 mM EDTA, 150 mM NaCl, 1% Triton X-100, 1 mM NaVO_3_, 1 mM NaF, 10 mM *β*-glycerophosphate, 1 mM phenylmethylsulfonyl fluoride, and 1.2 mg/mL cOmplete Lysis-M (Roche, Mannheim Germany) protease inhibitor cocktail]. The protein extracts were forced through a 22-gauge needle 10 times and centrifuged for 10 min at 14,000 rpm at 4°C, and the protein concentration was determined by the Pierce BCA Protein Assay kit (Thermo Fisher Scientific, Waltham, MA, USA). Approximately 25 *μ*g of protein was separated by 10% SDS-PAGE and transferred to nitrocellulose membranes and then incubated with the following primary antibodies: anti-phospho-Akt (sc-7985-R; Santa Cruz Biotechnology), anti-Akt (P-2482; Sigma-Aldrich), anti-phospho-ERK1/2 [pTpY185/187], (44-680G; Thermo Fisher Scientific), anti-ERK1/2 (sc-135900; Santa Cruz Biotechnology), and antiactin (sc-1616; Santa Cruz Biotechnology). Immunodetection was performed using a chemiluminescent substrate system (EMD Millipore Immobilon Western). Densitometry analysis was performed using the software ImageJ version 1.45 (National Institute of Health, USA).

### 2.3. Scratch-Wound Assays

A549 cells were grown to confluence on 30 mm culture dishes. The cells were starved for 24 h in DMEM/F12 without FBS and treated for 2 h with 12 *μ*M mitomycin C to inhibit proliferation during the experiment. After starvation, the cells were scratch-wounded using a sterile 200 *μ*L pipette tip, washed twice with phosphate buffered saline (PBS) to remove suspended cells, and refed with DMEM/F12 in the presence of 5 *μ*M of ATRA with or without 25 *μ*M of PD98059 for 48 h. Then, the cells were fixed with 4% paraformaldehyde in PBS for 15 minutes at room temperature, washed two times with PBS, and stained with 0.2% crystal violet for 15 minutes. The plates were left to dry for a day after washing with distilled water. To visualize the progress of cell migration into the wound, images were acquired with an Axiovert 40 CFL fluorescence microscope (Carl Zeiss AG, Oberkochen, Germany) using a 40x objective.

### 2.4. Quantification of Wound Size

The software ImageJ version 1.45 was used to document the width of the remaining gap at the end of 48 h. Each wound was measured five times, and the average was taken. Each experimental condition was repeated three times.

### 2.5. TUNEL Assay

Detection of apoptosis was performed using the DeadEnd Colorimetric TUNEL assay kit (Promega, Madison, WI, USA) according to the manufacturer's instructions. Briefly, A549 cells were grown on coverslips precoated with poly-L-lysine and were serum-starved and treated or nontreated (NT) for 48 h with 5 *μ*M of ATRA with or without 25 *μ*M of PD98059. After treatment, the cells were fixed with 4% paraformaldehyde in PBS and permeabilized with 0.2% Triton X-100 in PBS. The cells were incubated with recombinant terminal deoxynucleotidyl transferase (rTdT) and biotinylated nucleotides. Endogenous peroxidases were blocked with 0.3% hydrogen peroxide in PBS. The cells were incubated with streptavidin-HRP, which binds to biotinylated nucleotides incorporated at the 3′-OH DNA ends present in apoptotic cells. Streptavidin-HRP-labeled cells were detected by hydrogen peroxide and diaminobenzidine.

### 2.6. Proliferation Assay

A549 cells were seeded in a 96-well plate at a concentration of 10,000 cells/well in 100 *μ*L of DMEM/F12. The cells were treated for 48 h with 5 *μ*M of ATRA with or without 25 *μ*M of PD98059. Cell proliferation was measured using the 5-bromo-2′-deoxyuridine (BrdU) enzyme-linked immunosorbent assay (Roche) according to the manufacturer's instructions. For the last 2 h of the 48 h treatment period, the cells were pulsed with BrdU. Absorbance at 370 and 492 nm was measured in an Infinite M1000 plate reader (Tecan Group Ltd., Männedorf, Switzerland).

### 2.7. Statistical Analysis

The statistical significances of the differences among the data were determined by analysis of variance and the Newman-Keuls test or *t*-test, using GraphPad Prism 5.0 software (San Diego, CA, USA) when appropriate. *P* < 0.05 was considered statistically significant. Values are presented as the means ± SEM.

## 3. Results

### 3.1. ATRA Promotes ERK Activation by a Transcription-Independent Mechanism in Lung Adenocarcinoma Cell Line A549

**Figure 1:**
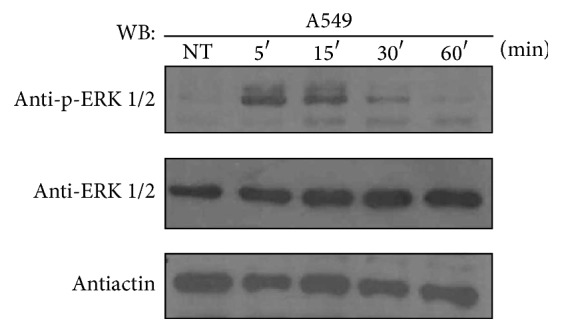
ATRA activates the ERK pathway through nongenomic mechanisms in A549 cells. A549 cells were serum-starved for 18 h, treated or nontreated (NT) with 5 *μ*M of ATRA for the times indicated. The phosphorylated form of ERK and total proteins were detected by western blot using specific antibodies.

**Figure 2:**
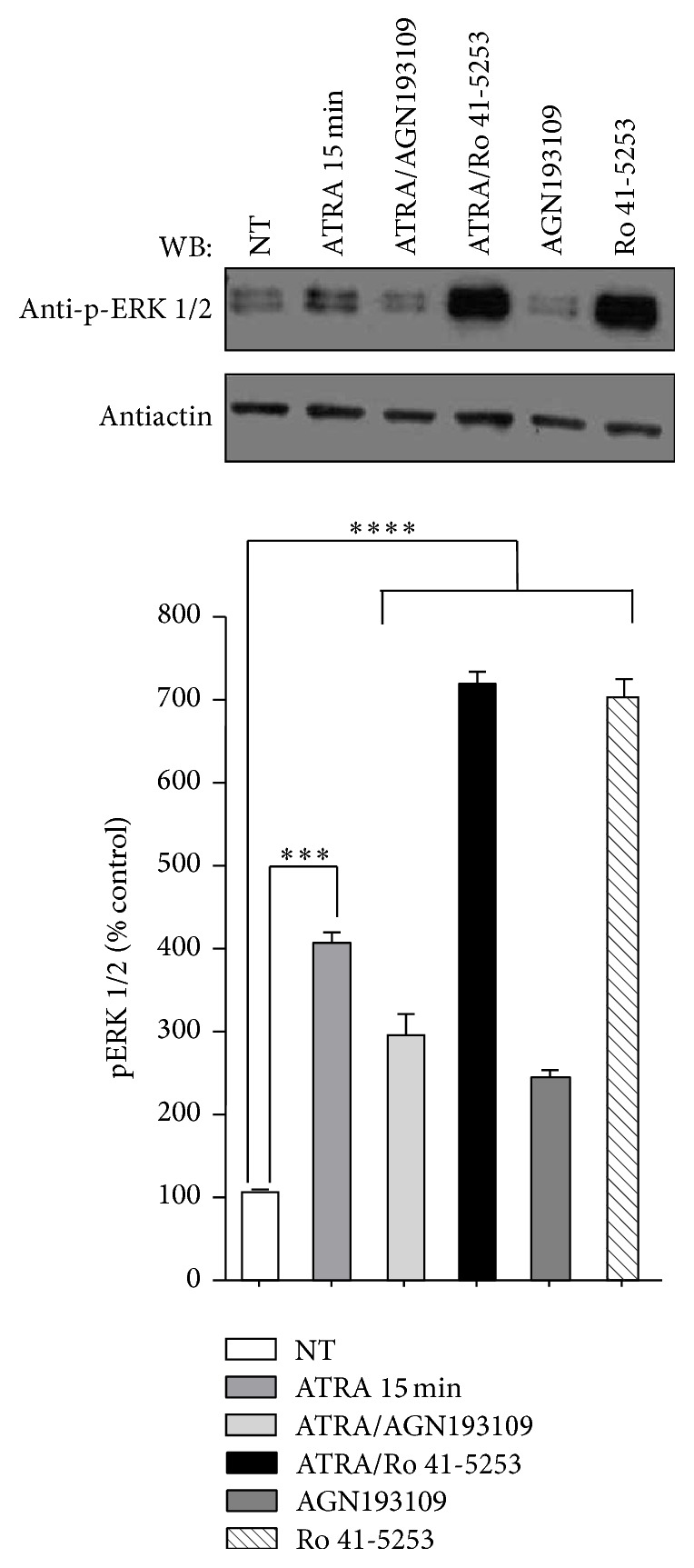
Effect of AGN193109 and Ro 41-5253 inhibitors on ATRA-induced ERK activation. A549 cells were serum-starved for 18 h, treated or nontreated (NT) with 5 *μ*M of ATRA for 15 minutes. Cells were preincubated for 1 h with 10 *μ*M of AGN193109 or 20 *μ*M of Ro 41-5253 alone or in combination with ATRA. The phosphorylated form of ERK was detected by western blot using specific antibodies. *β*-Actin was used as the loading control. The graph represents the densitometric values of ERK phosphorylation in three independent experiments (means ± SEM, ^***^ *P* < 0.0001; ^****^ *P* < 0.00001 compared with NT cells, analysis of variance and Newman-Keuls test).

We investigated the effects of ATRA on the regulation of the ERK pathway at different times in the ATRA-resistant A549 cell line. Our results show that treatment with ATRA caused a rapid phosphorylation of ERK, with a maximum effect at 5 and 15 min and a decline after 60 min (Figure 1). To confirm that ATRA promotes ERK activation by transcription-independent mechanism, we explored the effect of the pharmacologic inhibition of RARs. We used the selective RAR*α* antagonist Ro 41-5253, which binds RAR*α* and prevents the transcriptional activation of RAR*α* target genes [16] and AGN193109, a pan-retinoic acid receptor antagonist [17]. The results show that pretreatment with the inhibitors and treatment with ATRA for 15 minutes did not prevent ERK activation (Figure 2), which confirms that the activation of ERK is a transcription-independent mechanism. Interestingly, treatment with Ro 41-5253 alone or in combination with ATRA increases ERK phosphorylation.

### 3.2. PI3K Downregulates ERK Activation through the ATRA/RAR*α* Signaling Complex

**Figure 3:**
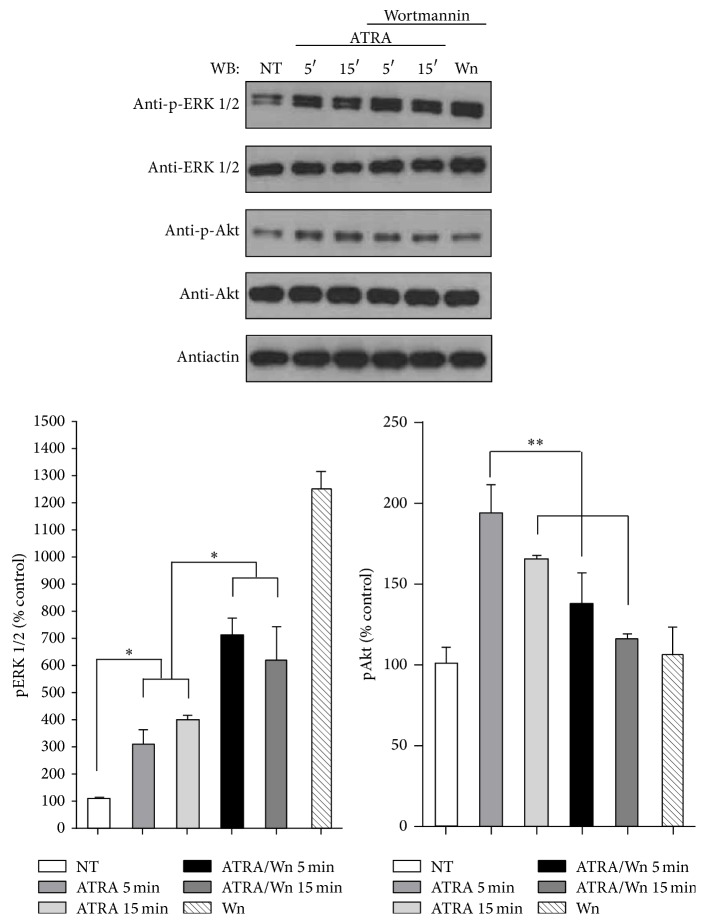
Effect of PI3K inhibitor wortmannin on ATRA-induced ERK phosphorylation. A549 cells were serum-starved for 18 h and treated or nontreated (NT) with 5 *μ*M of ATRA at different times. Cells were preincubated for 1 h with 10 *μ*M of wortmannin (Wm) alone or in combination with ATRA. The phosphorylated form of ERK and Akt and total proteins were detected by western blot using specific antibodies. The graph represents the densitometric analysis of ERK and Akt phosphorylation in three independent experiments (means ± SEM, ^*^ *P* < 0.05; ^**^ *P* < 0.001; analysis of variance and Newman-Keuls test).

Several feedback systems have been described in which inhibition of an intracellular pathway leads to activation of a parallel signaling pathway [18]. Previous results in our laboratory showed that ATRA promotes the formation of a signaling complex at the plasma membrane in a RAR*α*-dependent manner and activates the PI3K signaling pathway in the A549 cell line. Because ATRA promotes ERK activation in the same cell line and the activation of ERK is dependent on RAR*α*, we suspected that the ATRA/RAR*α* signaling complex at the plasma membrane might be modulating ERK phosphorylation through PI3K activation. To determine whether PI3K modulates ERK, we used an inhibitor of PI3K (wortmannin) in ATRA-treated cells at different times. Our results show that treatment with the PI3K inhibitor alone or in combination with ATRA increased the activation of ERK (Figure 3), suggesting that there is a crosstalk in these signaling pathways and PI3K negatively regulates ERK phosphorylation through the ATRA/RAR*α* signaling complex. The effectiveness of the wortmannin treatment was evaluated by testing its ability to counteract the phosphorylation of Akt in A549 cells. As expected, wortmannin prevented Akt phosphorylation (Figure 3).

### 3.3. The ERK Inhibitor Combined with ATRA Decreased Cell Proliferation

**Figure 4:**
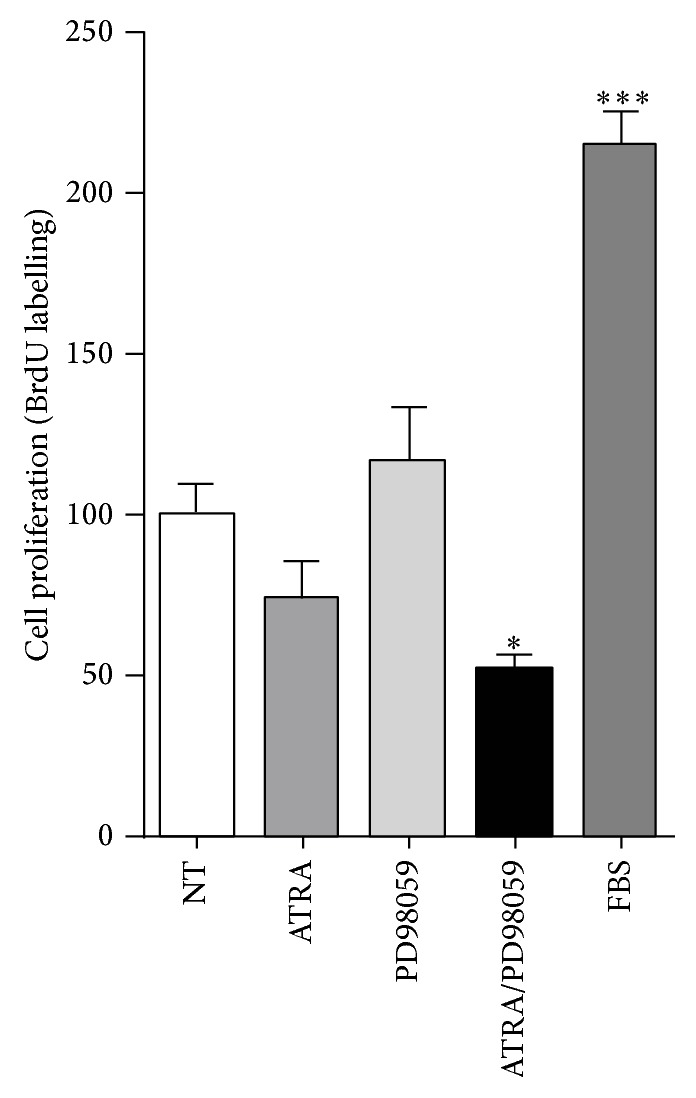
ERK activation is associated with proliferation on ATRA-resistant lung cancer cells. A549 cells were serum-starved and treated or nontreated (NT) with 5 *μ*M of ATRA alone or in combination with 25 *μ*M of PD98059 for 48 h. The proliferative effect was assessed by BrdU labeling according to the manufacturer's instructions. The graph shows the results of three independent experiments (means ± SEM, ^*^ *P* < 0.05; ^***^ *P* < 0.0001 compared with NT cells, analysis of variance, and Newman-Keuls test).

To examine the effect of ERK on cell proliferation induced by ATRA, A549 cells were treated for 48 h with ATRA and the pharmacological inhibitor of MEK-ERK, PD98059. As we reported previously, ATRA does not induce significant changes in proliferation. PD98059 treatment alone exerts no significant inhibitory effect on cell proliferation (Figure 4). However, the combination of ATRA with PD98059 decreases proliferation by 50%. These results suggest that activation of ERK is involved in blocking the classical antiproliferative effects of ATRA in the A549 cell line.

### 3.4. ATRA Promotes Cell Survival through the ERK Signaling Pathway

**Figure 5:**
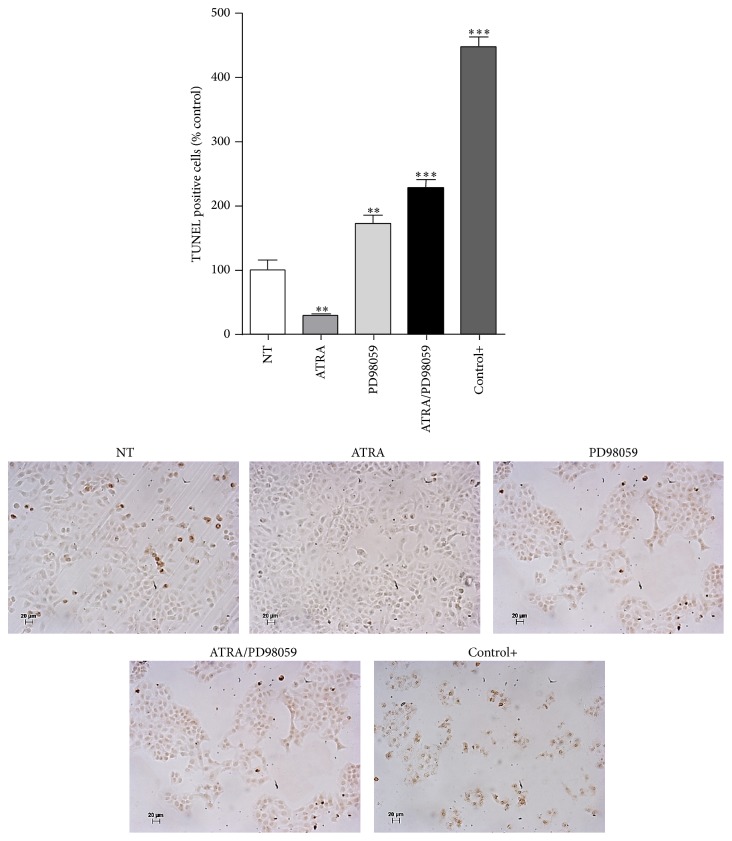
Pharmacologic inhibition of MEK-ERK in combination with ATRA promotes apoptosis. A549 cells were serum-starved and treated or nontreated (NT) with 5 *μ*M of ATRA alone or in combination with 25 *μ*M of PD98059 for 48 h. Cells irradiated with 20 J/m^2^ of UV light for 2 min were used as a positive control for apoptosis (+). DNA fragmentation was detected by TUNEL. The apoptotic cells are stained brown. Percentages of TUNEL-positive nuclei were quantified by counting 50 cells from five random microscopic fields (means ± SEM, ^**^ *P* < 0.001; ^***^ *P* < 0.0001 compared with NT cells, analysis of variance, and Newman-Keuls test).

Because ERK activity can promote cell survival [19], we tested whether activation of ERK mediated by ATRA is involved in survival. A549 cells were pretreated with the MEK-ERK inhibitor PD98059 and subsequently with ATRA for 48 h. Our results show an antiapoptotic effect mediated by ATRA, which can be avoided using the ATRA/PD98059 combination (Figure 5). These results indicate that ERK activation is also involved in the resistance to apoptosis of lung cancer cells treated with ATRA. Interestingly, treatment with PD98059 prevented Akt phosphorylation (Additional file 1: Figure S1 in the Supplementary Material available online at http://dx.doi.org/10.1155/2015/404368) suggesting that inhibition of MEK is enough to prevent the PI3K/Akt and ERK activation. Until now, any report had showed the involvement of MEK in the inhibition of the PI3K/Akt pathway in lung cancer cells.

### 3.5. ATRA Promotes Cell Migration by Activating ERK Signaling

**Figure 6:**
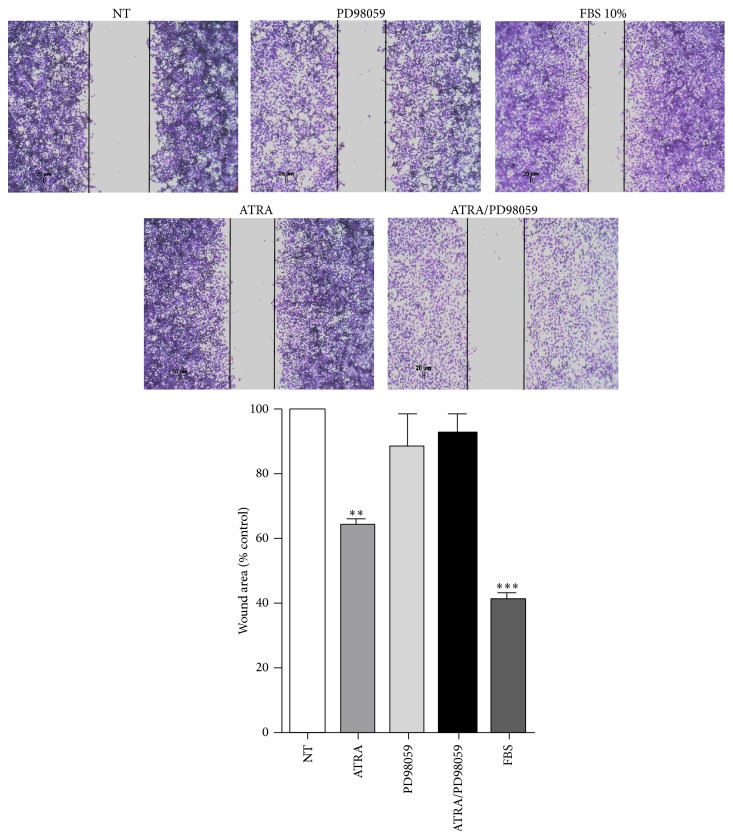
The inhibitor PD98059 with ATRA blocks the process of cell migration mediated by ATRA. A549 cells were serum-starved for 24 h and treated with 12 *μ*M mitomycin C for 2 h. After starvation, cells were scratch-wounded and treated or nontreated (NT) with 5 *μ*M of ATRA alone or in combination with 25 *μ*M of PD98059 for 48 h. Pictures were taken at 48 h after wounding and quantification of wound area was documented from five random microscopic fields (means ± SEM, ^**^ *P* < 0.001; ^***^ *P* < 0.0001 compared with NT cells, analysis of variance, and Newman-Keuls test).

The ERK signaling pathway has been previously implicated in cell migration. To determine the functional consequences of ERK activation by ATRA, we used an* in vitro* scratch wound-healing assay in the presence of the ERK inhibitor PD98059. The cells treated with ATRA healed more of the wounded area than the cells in the control condition (Figure 6), and these results demonstrate that ATRA promotes migration in A549 cells. However, when the cells were pretreated with the ERK inhibitor, the cells showed significant delays in wound closure induced by ATRA.

## 4. Discussion

The ERK signaling cascade is important in the regulation of diverse biological functions including cell survival, motility, and proliferation [20]. Aberrant activation of kinases in this pathway is frequently reported in human cancer. In this report, we show evidence of ERK phosphorylation within 5–15 minutes of ATRA treatment in A549 lung cancer cells, which serves as a measurable response of nongenomic activation. ATRA-induced ERK activation in lung cancer cells may be mediated via RAR*α* and PI3K. Extensive evidence suggests that RAR*α* associated with the plasma membrane facilitates the ATRA activation of kinase pathways. The existence of rapid, nongenomic ATRA signaling is incontrovertible and has been reported in various cell types, such as PC12, bronchial epithelial cells [12], and Sertoli cells [21]. To support our results, we used specific antagonists (AGN 193109 and Ro 41-5253) that prevent the expression of ATRA target genes but that do not prevent ERK activation by ATRA, which is consistent with other reports using a different antagonist in tracheobronchial cells [12]. Moreover, we observed that the RAR*α* antagonist (Ro 41-5253) increased ERK phosphorylation. It has been shown that Ro 41-5253 induces conformational change in RAR*α*, and this structural alteration decreases the interaction with other transcription factors [22]; this could mean that antagonist-induced conformational changes cause an increase in the affinity to components of the ERK pathway.

How ATRA activates ERK in lung cancer cells is still unknown; there are reports that demonstrate that RAR*α* mediates the rapid effects of ATRA in neuronal cells because it is present in membranes and activates ERK through the activation of the PI3K/Akt pathway [13]. RAR*α* localized in lipid rafts interacts with G*α*q proteins and activates Rho-GTPases, p38MAPK, and MSK1 [23]. Another report showed that RAR*γ* is present in cytoplasm and is involved in the nongenomic effects of ATRA in association with Src in the activation of ERK in neuronal cells [24]. Besides, ERK2 directly interacts with and phosphorylates RAR *β*2 in PC12 cells [25]. Another mechanism for the activation of ERK by ATRA might be related to its lipophilic nature; retinoids bind to regulatory domain of cRaf and PKC isoforms, modulating its activity [26]. These results indicate that different types of RAR may be involved in ERK activation depending on the cell type and the ability of ATRA to interact with protein targets in plasma membrane and cytosol may depend on its chemical structure and the affinity for certain domains. Nevertheless more investigation is needed to clarify the transcription-independent mechanism of ERK activation by ATRA. In a previous report, we demonstrated that, in lung cancer, RAR*α* is responsible for mediating the nongenomic effects of ATRA by forming a signaling complex that is able to activate PI3K within the first few minutes after treatment with ATRA. To investigate whether RAR*α* and PI3K are also involved in ERK activation by ATRA, we used a PI3K inhibitor (wortmannin), and we observed that PI3K activation by ATRA downregulated ERK activation within the first few minutes after the ATRA treatment and wortmannin had an enhancing effects on the ERK phosphorylation. The inhibition of PI3K with wortmannin has been shown to block activation of MAP kinase in some but not all cells, and the inhibition of the MAP kinase pathway by wortmannin is cell type- and ligand-specific [27]. Consistent with our results, other groups have shown that inhibition of the PI3K/Akt/mTORC1 pathway leads to activation of the Ras/MEK/ERK signaling cascade in lung cancer cells, and this is dictated by the K-Ras and c-Met status and the protein phosphatase activity [28]. In addition, other groups have shown that inhibitors of signaling pathways such as PI3K, AKT, and mTOR actually lead to activation of MEK/ERK signaling in many cancer types [29, 30]. Recent reports showed that PI3K inhibitors induce increase ERK phosphorylation in a dose-dependent manner in breast cancer cells in which the activation of HER family receptors is involved in the ERK signaling. Moreover, block PI3K highlights the role of RAS/RAF/MEK/ERK pathway as an escape mechanism which is activated in response to PI3K inhibition. Mutations of RAS and RAF family members are frequently found in human tumors and they are responsible for promoting resistance to PI3K inhibitors; this effect is reverted by the combination of MEK and PI3K inhibitors in mouse models of mutated KRAS lung cancer resulting in synergistic tumor shrinkage [31]. On the other hand, it has been reported that MEK inhibition leads to PI3K/Akt activation; Turke et al. demonstrated that this interplay is through a negative feedback on ErbB receptors; in the presence of a MEK inhibitor, ERK is inhibited and the T669 of EGFR a MAPK target site is blocked, increasing EGFR and ErbB3 tyrosine phosphorylation and upregulating the PI3K/Akt activation in lung cancer cells [18]. Our results showed a crosstalk between PI3K/Akt and ERK since treatment with wortmannin and ATRA was able to increase the ERK phosphorylation and conversely the MEK inhibitor avoids the AKT phosphorylation in lung cancer cells. There are no reports about RAR*α* participation in nongenomic ERK activation by ATRA, and our results demonstrate that PI3K activation downregulates ERK activation through the ATRA/RAR*α* signaling complex, which is responsible for mediating the rapid effects in lung cancer cells.

To determinate whether ATRA has an effect on cell proliferation through ERK activation in lung cancer cells, we evaluated proliferation in cells treated with ATRA for 48 hours. Our results show that treatment with ATRA does not have an inhibitory effect in this cell line, which is consistent with other reports that ATRA does not exert an inhibitory effect in some lines that are resistant to the treatment [32, 33]. However, we were able to demonstrate that a combined treatment of ATRA with the MEK inhibitor decreased cell proliferation by 50 percent, which suggests that inhibition of the MAPK pathway is a key point for the promotion of the inhibitory effect of ATRA on cell proliferation.

Cell survival is another important process regulated by the ERK pathway. In a previous study, we demonstrated that treatment with ATRA results in a tendency to promote survival in the A549 cell line; interestingly, our results suggest that the ERK pathway is involved in cell survival mediated by ATRA. The addition of the MEK inhibitor counteracts the survival mechanism mediated by ATRA, increasing the number of apoptotic cells. The action of the MEK inhibitor involves promoting cell cycle arrest; increasing the percentage of cells in G1 phase, an indicator of apoptosis; and decreasing the expression of cyclin E, cyclin D1, and pro-caspase 3 [34, 35]. These mechanisms might be responsible for our results; we observed an increase in the number of apoptotic cells with the MEK inhibitor alone, and we observed an even higher number when we used the combination treatment with ATRA. This result shows that ATRA is effective in promoting apoptosis when the ERK pathway is inhibited.

The functional effect of ATRA on cell migration and the molecular mechanism to promote it are unknown. We evaluated whether the ERK signaling pathway could participate in this process, and our results showed that treating cells with ATRA for 48 hours promotes cell migration in lung cancer cells, which suggests that ATRA induces cytoskeletal reorganization in the cells to facilitate migration through an ERK-dependent mechanism. This behavior had not been previously demonstrated, although some reports indicate that retinoids are involved in the expression of metalloproteases [36], which would help degrade the extracellular matrix of the cells to facilitate migration to and invasion of other tissues. Our results strongly suggest that MEK-ERK pathway inhibition may be able to halt the migration process and that the ERK pathway may mediate the effects of ATRA on migration. In 2005 and 2013, it was shown that retinoic acid activates the PI3K pathway, causing Rac1 activation, suggesting that the activation of PI3K and Rac1 also regulates MAPK activation [11, 37]. Rac1 has been implicated in the regulation of cell migration, inducing reorganization of the actin cytoskeleton to form membrane ruffles in fibroblasts and regulating the expression of several matrix metalloproteases [38]. Our results suggest that treatment with ATRA promotes migration in lung cancer cells mediated by activation of the ERK signaling pathway.

Our results propose using MAPK inhibitors combined with ATRA to reverse the resistance to ATRA in lung cancer cells, thereby inhibiting cell proliferation, promoting apoptosis, and avoiding the cell migration process caused by ATRA.

## 5. Conclusions

ATRA activates the ERK signaling pathway by a transcription-independent mechanism, and this mechanism involves the RAR*α* receptor signaling pathway and PI3K downregulation of ERK activation. ATRA promotes cell survival and migration through activation of the ERK pathway in lung adenocarcinoma cells; in contrast, inhibition of the ERK pathway in combination with ATRA decreased cell proliferation. The use of a combination treatment of ATRA and inhibitor of ERK could potentially be used as a treatment to reduce proliferation, promote apoptosis, and prevent the process of cell migration induced by ATRA in patients with lung cancer.

## Supplementary Material

FIGURE S1. Effect of MEK inhibitor PD98059 on ATRA-induced Akt activation. A549 cells were serum-starved for 18 h, treated or non-treated (NT) with 5 µM of ATRA for 15 minutes alone or in combination with 25 µM of PD98059 for 90 minutes. The phosphorylated form of Akt and total proteins were detected by western blot using specific antibodies. β-Actin was used as the loading control. The graph represents the densitometric analysis of Akt phosphorylation in three independent experiments (means ± SEM, ∗p< 0.05; ∗∗p< 0.001 compared with NT cells; analysis of variance and Newman-Keuls test).
