# Supplementary material for: All-Trans Retinoic Acid Induces Proliferation, Survival, and Migration in A549 Lung Cancer Cells by Activating the ERK Signaling Pathway through a Transcription-Independent Mechanism
Source: Biomed Res Int. 2015 Oct 18;2015:404368. doi: 10.1155/2015/404368 (PMC4628773; doi:10.1155/2015/404368)
Supplement: Supplementary file 1 — FIGURE S1. Effect of MEK inhibitor PD98059 on ATRA-induced Akt activation. A549 cells were serum-starved for 18 h, treated or non-treated (NT) with 5 µM of ATRA for 15 minutes alone or in combination with 25 µM of PD98059 for 90 minutes. The phosphorylated form of Akt and total proteins were detected by western blot using specific antibodies. β-Actin was used as the loading control. The graph represents the densitometric analysis of Akt phosphorylation in three independent experiments (means ± SEM, ∗p< 0.05; ∗∗p< 0.001 compared with NT cells; analysis of variance and Newman-Keuls test). [file 404368.f1.pdf]

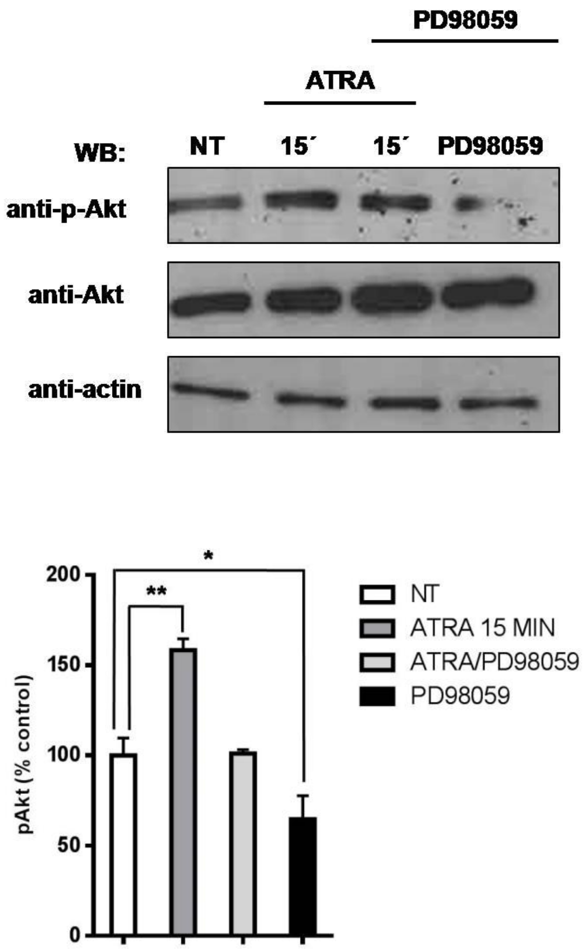

FIGURE S1. Effect of PD98059 inhibitor on ATRA-induced Akt activation. A549 cells were serum-starved for 18 h, treated or non-treated (NT) with 5 μM of ATRA for 15 minutes. Cells were preincubated for 90 minutes with 25 μM of PD98059 alone or in combination with ATRA. The phosphorylated form of Akt and total proteins were detected by western blot using specific antibodies. β-Actin was used as the loading control. The graph represents the densitometric values of Akt phosphorylation in three independent experiments (means ± SEM, *$p < 0.05$; **$p < 0.001$ compared with NT cells, analysis of variance and Newman-Keuls test).
